# Prevention and treatment of respiratory syncytial virus bronchiolitis and postbronchiolitic wheezing

**DOI:** 10.1186/rr183

**Published:** 2002-06-24

**Authors:** Jan LL Kimpen

**Affiliations:** 1 Wilhelmina Children's Hospital, University Medical Center Utrecht, Utrecht, The Netherlands

**Keywords:** bronchiolitis, lower respiratory tract infection, palivizumab, reactive airway disease, respiratory syncytial virus

## Abstract

Respiratory syncytial virus (RSV) is the primary cause of hospitalization for acute respiratory tract illness in general and specifically for bronchiolitis in young children. The link between RSV bronchiolitis and reactive airway disease is not completely understood, even though RSV bronchiolitis is frequently followed by recurrent episodes of wheezing. Therapy with ribavirin does not appear to significantly reduce long-term respiratory outcome of RSV lower respiratory tract infection, and corticosteroid or bronchodilator therapy may possibly improve outcomes only on a short-term basis. No vaccine against RSV is yet available. It is not known whether prophylaxis with RSV intravenous immune globulin or palivizumab can reduce postbronchiolitic wheezing.

## Introduction

Respiratory syncytial virus (RSV) can cause acute clinical findings that range from symptoms of upper respiratory infection, with or without otitis media, to severe lower respiratory tract disease. Virtually all children become infected with RSV within 2 years of birth, most commonly before 6 months of age, and 1% require hospitalization [1]. RSV is the primary cause of hospitalization for acute respiratory tract illness in young children, and it may be responsible for 40–90% of cases of bronchiolitis, for 5–40% of cases of pneumonia, and for 10–30% of cases of tracheobronchitis in this age group [2].

RSV is transmitted most commonly by direct contact with surfaces or persons contaminated with infectious nasal secretions. After an incubation period of 2–8 days, RSV replicates in the nasopharyngeal epithelium and can spread to the lower respiratory tract within 1–3 days [3]. Pneumonia or bronchiolitis occurs in 30–71% of infants and young children at the time of first RSV infection [4].

RSV bronchiolitis is often followed by recurrent episodes of wheezing, although the pathogenesis of this link is poorly understood. Bont *et al.*[5] found that 47% of 130 infants hospitalized for RSV lower respiratory tract infection (LRTI) developed recurrent wheezing during a 1-year follow up. The occurrence of wheezing was significantly higher in infants with airflow limitation (61%) than for those without airflow limitation (21%; *P* < 0.001) during the acute infection. In a study by conducted by Stein *et al.*[6], 207 children who had RSV LRTI before age 3 years also had a significantly increased risk for both infrequent wheezing and frequent wheezing by age 6 years (3.2 and 4.3 times greater incidence than in children who did not have RSV LRTI before age 3 years, respectively; *P* < 0.001). However, this risk decreased and was no longer significant by age 13 years, and none of the patients was hospitalized. Sigurs *et al*. [7] demonstrated that RSV bronchiolitis that was severe enough to require hospitalization in 47 infants was highly associated (*P* < 0.001) with development of asthma and recurrent wheeze up to age 7.5 years. The cumulative prevalence of asthma was 30% in the RSV group and 3% in the control group (*P* < 0.001), and the cumulative prevalence rates of any wheezing were 68% and 34%, respectively (*P* < 0.001).

The majority of children who experience postbronchiolitic wheezing have multiple (recurrent) episodes. Hall [3] cited subsequent episodes of wheezing in 40–50% of infants hospitalized with RSV bronchiolitis, imposing a significant burden on health care resources. During the 1980s, an estimated 100,000 children were hospitalized with RSV infection in the USA annually [3,8], at a cost of US$300 million. In the study conducted by Bont *et al*. [5] recurrent wheezing in the hospitalized infants was defined as two or more episodes; respiratory symptoms were recorded in a daily diary. Infants had a total of 241 episodes of wheezing, with a median number of two episodes.

It is difficult to predict which children will develop recurrent wheezing, although some clinical and immunologic parameters may be useful in prediction models in the future. Airflow limitation during RSV LRTI is the first useful clinical predictor of subsequent recurrent wheezing and physician diagnosed asthma in early childhood [5]. Another predictor may be monocyte interleukin-10 responses *in vitro* to stimulation with nonspecific stimuli [9]. In addition, a high (≥ 16 μg/l) serum eosinophil cationic protein concentration during the acute phase of bronchiolitis is a specific but insensitive predictor of wheezing after bronchiolitis [10]. Thus, a high serum level of eosinophil cationic protein is strongly predictive of subsequent wheezing, but low values do not exclude wheezing episodes in the future.

The pathogenesis of recurrent wheezing after RSV LRTI is not completely understood. Prospective, randomized studies are needed to determine whether severe RSV LRTI causes long-term pulmonary sequelae and a predisposition to wheezing, or whether inherent genetic or structural abnormalities predispose a child to both severe LRTI and wheezing later in life. If severe RSV LRTI causes subsequent wheezing, then reducing the severity of RSV LRTI should reduce the risk for subsequent wheezing. This issue has been explored in several studies of RSV LRTI treatment or prevention; these studies are discussed below.

## Therapy for respiratory syncytial virus bronchiolitis

Antiviral therapy with aerosolized ribavirin and symptomatic therapy with bronchodilators and corticosteroids are the available treatment options for RSV infections. Unfortunately, they produce only a modest short-term improvement in respiratory outcome, or have no effect at all.

Ribavirin is a synthetic guanosine analog and broad-spectrum antiviral agent that is approved only for hospitalized infants and young children with severe RSV infection. Ribavirin inhibits RSV replication during the active replication phase. Use of aerosolized ribavirin has been associated with improved oxygenation and clinical scores [11] and with reduced levels of secretory mediators of inflammation associated with severe disease and wheezing [12]. However, some investigations were criticized for their methodology and use of subjective end-points (e.g. clinical score) rather than objective end-points (e.g. mortality, arterial oxygen saturation, and need for or duration of mechanical ventilation) [13]. Any beneficial effects of ribavirin, such as a reduction in duration of mechanical ventilation or hospitalization, are unproven [3], and routine use in high-risk children with RSV infections is no longer warranted [13].

Most available data do not support the use of corticosteroids in the treatment of acute RSV bronchiolitis. Intravenous hydrocortisone followed by oral prednisone [14], intramuscular or oral dexamethasone [15,16], and oral prednisolone [17] have yielded little or no benefit, except possibly in a select subgroup of patients.

Several trials [18-21] have also shown that β_2_-agonist bronchodilators, such as salbutamol and the anticholinergic agent ipratropium bromide, have no beneficial effect on acute RSV bronchiolitis. Although bronchodilators may produce a modest short-term improvement in clinical scores [13], Flores and Horwitz [22] concluded (on the basis of an extensive meta-analysis) that there is insufficient evidence to support the use of β_2_-agonists to treat bronchiolitis.

Studies of the efficacy of vitamin A [23] and interferon [24] also had disappointing results. Oral administration of vitamin A did not decrease respiratory morbidity in children with acute RSV bronchiolitis [23]. In a double-blind, placebo-controlled study [24], IFN-α-2a had a prophylactic effect but did not reduce the severity of signs and symptoms or duration of illness. There was a significant difference (*P* < 0.05) in the frequency of colds in the two groups: one out of 19 receiving IFN-α-2a and seven out of 19 receiving placebo had colds. Also, 14 out of 19 receiving placebo showed laboratory evidence of RSV infection, versus 10 of 19 receiving IFN-α-2a. Because the risk for a secondary bacterial infection in patients with RSV bronchiolitis is very low [25], routine use of antibiotics is not warranted.

The results of two therapeutic trials with RSV intravenous immune globulin (RSV-IGIV; RespiGam™, MedImmune, Inc., Gaithersburg, MD, USA) were also disappointing. RSV-IGIV was no more effective than placebo in previously healthy children hospitalized with RSV infection with regard to duration of stay either in hospital or on the intensive care unit [26], or was it beneficial in hospitalized children at high risk for severe RSV infection [27]. However, RSV-IGIV has been shown to be effective in the prevention of RSV LRTI in these high-risk children.

## Prophylaxis for respiratory syncytial virus bronchiolitis

Prevention of RSV bronchiolitis has met with greater success than has treatment. Although no vaccine is available to prevent RSV bronchiolitis, passive immunization can be conferred by RSV-IGIV, which contains high levels of RSV neutralizing antibody, or by intramuscular injections of the humanized monoclonal antibody palivizumab (Synagis^®^; MedImmune, Inc.). Both products are costly, however.

### Vaccines

The development of vaccines for RSV has been confounded by failure to achieve durable immunity, even after natural infection, and by an incomplete understanding of the immune response to RSV. In the 1960s, a formalin-inactivated aluminum-precipitated vaccine failed to protect infants and children against naturally acquired RSV infection. Furthermore, on rechallenge with RSV the vaccine induced an immune-mediated response, resulting in increased hospitalizations for lower respiratory tract pathology and increased mortality [28-30].

Even if an effective vaccine could be developed that protects for at least the first few years of life, it might not be available for general use for another 5–10 years. Vaccination research has produced such candidates as the recombinant vaccine BBG2Na; subunit vaccines such as the purified fusion protein-2; and cold-passaged, temperature-sensitive vaccines. However, phase III efficacy trials in infants, young children, and the elderly are still lacking. In a phase I trial, the recombinant fusion protein BBG2Na was highly immunogenic and well tolerated, and had few side effects in healthy volunteers [13]. The use of purified fusion protein-2, which contains fusion protein of approximately 98% purity and small quantities of other virus proteins [31], resulted in a good immune response and a significant reduction in LRTIs in RSV-seropositive children with cystic fibrosis during the RSV season [32]. Important side effects, including upper respiratory tract symptoms such as nasal congestion, were observed with several candidate attenuated vaccines, particularly in infants younger than 2 months [13].

### Respiratory syncytial virus immune globulin intravenous

Passive immunization with RSV-IGIV is effective as long as the neutralizing antibody titer is adequate. For example, in a study conducted by Groothuis *et al.*[33], 249 high-risk infants and young children with or without chronic lung disease and those with congenital heart disease were randomized to receive monthly infusions of high-dose RSV-IGIV (750 mg/kg body weight, *n* = 81), low-dose RSV-IGIV (150 mg/kg body weight, *n* = 79), or no prophylaxis (*n* = 89). The high-dose group showed a significant reduction (62%; *P* = 0.01) in the incidence of all RSV LRTIs and a 72% reduction (*P* = 0.03) in moderate or severe RSV LRTIs. Furthermore, the high-dose group had significantly fewer hospitalizations and hospital days (63%; *P* = 0.02), fewer days in the intensive care unit (97–100% reduction for both high and low doses; *P* = 0.05 for high dose and *P* = 0.03 for low dose), and decreased use of ribavirin (9%; *P* = 0.05). The greatest benefit of RSV-IGIV was observed in preterm infants with or without bronchopulmonary dysplasia (BPD). In a sub-analysis of that study [34], high-dose RSV-IGIV significantly reduced (*P* = 0.006) the incidence of moderate to severe RSV LRTIs and also decreased RSV hospitalizations in preterm infants with or without BPD (*n* = 58) versus placebo (*n* = 58; *P* = 0.06).

The PREVENT trial [35] found that monthly high-dose RSV-IGIV (750 mg/kg, *n* = 250) in infants with prematurity and BPD was associated with a 41% reduction in hospitalizations and 53% fewer days of hospitalization for RSV as compared with the placebo group (*n* = 260). The number of patients requiring mechanical ventilation, however, did not differ between the two groups. There was also no difference in the proportion of children who reported adverse events.

Drawbacks associated with the use of RSV-IGIV are the long duration of intravenous administration (several hours), the considerable volume needed (15 ml/kg), possible interference with normal vaccinations, and high cost. RSV-IGIV is not recommended in infants with cyanotic congenital heart disease because it was associated with a high rate of adverse events.

### Palivizumab

Palivizumab is an IgG_1_ humanized monoclonal antibody that exhibits neutralizing and fusion-inhibitory activity against RSV. In the randomized, double-blind, multicenter IMpact-RSV Trial [36], monthly palivizumab prophylaxis by intramuscular injection (15 mg/kg body weight, *n* = 1002) versus placebo (*n* = 500) resulted in a significant reduction (55%; *P* = 0.00004) in RSV-related hospitalization in high-risk premature infants or infants with BPD. During the 150-day follow up, premature infants without BPD benefited most from the therapy. Palivizumab was safe and well tolerated, and there were no serious side effects or significant differences between the placebo and treatment groups in adverse events.

The advantages of palivizumab are its easy administration and lack of interference with normal vaccinations. As a result, it is generally used more commonly than RSV-IGIV. Its major disadvantage is its high cost. Other IgG monoclonal antibodies are being studied that may be future candidates for phase III clinical trials of prevention of RSV infection in high-risk infants.

## Prevention of recurrent wheezing episodes

If the recurrent wheezing episodes associated with severe RSV LRTIs in infants are in fact due to the initial RSV infection, then these sequelae might be prevented in one of two ways. One is treating the initial RSV bronchiolitis episode in order to lessen its severity; treatment options include ribavirin and corticosteroids. The other option is to prevent RSV bronchiolitis; effective options include RSV-IGIV and palivizumab [37].

### Ribavirin

Several studies have shown that ribavirin therapy for RSV LRTI appears to have no significant effect on long-term respiratory outcomes, although it may affect short-term outcomes. In the first study of ribavirin and its long-term sequelae in children hospitalized with RSV LRTI, Krilov *et al.*[38] identified no difference in the incidence of reactive airway disease (RAD) or in pulmonary function 6 years after treatment in those who received ribavirin (*n* = 33) and age-matched control children (*n* = 67). In that study, the ribavirin group had more severe disease. Similarly, a 9-year follow-up study of children hospitalized for RSV LRTI conducted by Long *et al.*[39] suggested that those randomized to receive ribavirin (*n* = 28) did not have a better outcome than did those randomized to receive placebo (*n* = 26). There were no differences between the two groups for any of the parameters studied, including recurrent LRTI, wheezing, and pulmonary function.

In a 7-year follow-up study, Rodriguez *et al.*[40] found no difference in RAD, wheezing, or pneumonia between children who received ribavirin (*n* = 24) and those who received placebo (*n* = 11), and no difference in results of methacholine challenge tests. However, none of six placebo patients had normal or mildly abnormal pulmonary function test results as compared with seven out of 13 ribavirin-treated patients. In contrast, the results of a 1-year retrospective study [41] suggest that ribavirin therapy might affect the short-term outcome of severe RSV bronchiolitis. Infants who received ribavirin (*n* = 22) had a significant reduction (*P* < 0.05) in the prevalence of RAD as compared with those who underwent conservative management (*n* = 19), both in terms of percentage of patients developing airway reactivity (59% and 89%, respectively) and number of episodes of RAD (31 and 70, respectively).

### Corticosteroids

Corticosteroids to reduce local inflammation and bronchodilators to relax airway smooth muscle at the time of acute RSV infection were considered for several reasons [42]. First, there is a striking similarity between the clinical syndromes of bronchiolitis and childhood asthma, for which bronchodilators and corticosteroids are the mainstays of treatment. Second, RSV bronchiolitis is often followed by recurrent episodes of wheezing. Finally, bronchiolitis and childhood asthma have pathophysiologic and immunopathogenic mechanisms in common. The available data fail to show that corticosteroid therapy during or after RSV bronchiolitis effectively prevents recurrent wheezing. Only four out of the 10 studies [43-52] shown in Table 1 demonstrated any positive effect. In addition, the prevalence of postbronchiolitic wheezing was not reduced by oral prednisone with nebulized budesonide during the acute infection [52]. Although there are some indications that corticosteroids may have an effect in patients with more severe infection [53], longer-term studies, especially those that include pulmonary function testing, are needed.

### Respiratory syncytial virus immune globulin intravenous

In a study conducted at the University of Colorado School of Medicine and Children's Hospital, Wenzel *et al*. [54] investigated the use of RSV-IGIV to prevent recurrent wheezing. Those investigators found that RSV-IGIV prophylaxis may have long-term effects on clinical and immunologic parameters of recurrent wheezing.

### Palivizumab

Currently, no data are available on the use of palivizumab for treatment or prevention of bronchiolitis, or prevention of recurrent wheezing, although one study is underway (Protocol W00-353; Abbott Laboratories, Abbott Park, IL, USA). Children who have received palivizumab will be compared with children who have had documented RSV bronchiolitis and control children. Approximately 100 children will be enrolled in each cohort. They will be followed for 3 years by means of monthly telephone calls and office visits at 6, 12, 24, and 36 months. Outcomes will consist of a respiratory questionnaire, incidence of wheezing, medications used, hospitalizations, and pharmacoeconomic evaluation. Results are expected in late 2003.

## Conclusion

Studies have shown that ribavirin therapy appears to have no significant effect on reducing the long-term respiratory outcomes associated with RSV LRTI. Inhaled corticosteroid or bronchodilator therapy administered at the time of acute RSV infection may improve respiratory outcomes only in the short term. RSV-IGIV and palivizumab are effective agents for prevention of RSV LRTI in children with BPD and/or prematurity, but their long-term effect on the development of asthma and recurrent wheezing is not clear. Further research is needed to elucidate the mechanisms involved and to evaluate the impact of available therapies on long-term respiratory outcomes of RSV LRTI.

## Abbreviations

BPD = bronchopulmonary dysplasia; IFN = interferon; LRTI = lower respiratory tract infection; RAD = reactive airway disease; RSV = respiratory syncytial virus; RSV-IGIV = respiratory syncytial virus immune globulin intravenous (human).

## Figures and Tables

**Table 1:** Effect of corticosteroids on recurrent wheezing

Reference	Effect	Children treated/control children (*n*)	Proven RSV infection (*n*)	Corticosteroid or other drug therapy	Duration of follow-up	Comments
[43]	Positive	68/120	All	Salbutamol, epinephrine, betamethasone, terbutaline, budesonide	24 months	Group 2 (86% received corticosteroids after discharge) versus group 1 (13% with persistent symptoms received drug after discharge)
[44]	Positive	40/41	All	Budesonide for 1 week or 8 weeks	2 years	Fewer patients in 1-week group (18%) and 8-week group (12%) had asthma versus control group (37%; *P* < 0.02)
[45]	Positive	Cromolyn 34; budesonide 34; controls 32	21	Budesonide; cromolyn sodium	16 weeks	Fewer wheezing episodes in last 8 weeks (cromolyn 19%, budesonide 16%, controls 47%; *P* < 0.05)
[46]	Positive	20/21	Not identified	Beclomethasone dipropionate	12 weeks	Wheezing (*P* = 0.005) and sleep patterns (*P* < 0.005) significantly improved in treated group at 6 weeks
[47]	None	83/82	All	Budesonide	12 months	No differences in duration of hospital admission, time to become symptom free, readmission rates, general practitioner consultation rates, or use of antiwheeze medication during follow up
[48]	None	Cromolyn 29; budesonide 31; controls 29	23	Budesonide; cromolyn sodium	3 years	Therapy for 4 months did not affect occurrence of asthma at 3 years
[49]	None	27/27	All	Prednisolone	3–7 years	No difference in number of wheezers by age 5 years
[50]	None	26/28	35	Budesonide	12 months	No difference in hospital admissions or increase in symptoms at 1 year
[51]	None	20/18	50% in each group	Prednisone	2 years	Similar prevalence of chronic respiratory symptoms at 2 years
[52]	None	21/19	83%	Budesonide	6 months	No difference in prevalence of wheeze, cough/wheeze scores, or bronchodilator use

RSV, respiratory syncytial virus.
